# Investigation of 2D Rainbow Metamaterials for Broadband Vibration Attenuation

**DOI:** 10.3390/ma13225225

**Published:** 2020-11-19

**Authors:** Han Meng, Dimitrios Chronopoulos, Nick Bailey, Lei Wang

**Affiliations:** 1 Institute for Aerospace Technology & The Composites Group, University of Nottingham, Nottingham NG8 1BB, UK; dimitrios.chronopoulos@nottingham.ac.uk (D.C.); nickbailey999@gmail.com (N.B.); 2 Department of Mechanical and Construction Engineering, University of Northumbria, Newcastle upon Tyne NE1 8QH, UK; 3 School of Mechanical Engineering, Xi’an Jiaotong University, Xi’an 710049, China; wlei292@xjtu.edu.cn

**Keywords:** 2D metamaterials, bandgaps, rainbow, experimental, additive manufacturing

## Abstract

Phononic crystals (PnCs) and metamaterials are widely investigated for vibration suppression owing to the bandgaps, within which, wave propagation is prohibited or the attenuation level is above requirements. The application of PnCs and metamaterials is, however, limited by the widths of bandgaps. The recently developed rainbow structures consisting of spatially varied profiles have been shown to generate wider bandgaps than periodic structures. Inspired by this design strategy, rainbow metamaterials composed of nonperiodic mass blocks in two-dimensional (2D) space were proposed in the present study. The blocks were connected by curved beams and tessellated with internal voids to adjust their masses. In order to demonstrate the effects of the rainbow design, two 2D metamaterials, with periodic and nonperiodic units, respectively, were investigated and manufactured using additive manufacturing technologies. Receptance functions, i.e., displacement frequency response functions, of the manufactured metamaterials were calculated with finite element models and measured with a testing system containing a mechanical shaker, an impedance head, and a laser Doppler vibrometer. The obtained numerical and experimental results showed that the metamaterial with rainbow blocks has extended bandgaps compared with the periodic metamaterial.

## 1. Introduction

Phononic crystals (PnCs) and elasto-acoustic metamaterials are artificial structures that possess exceptional properties that cannot be achieved by natural materials. It is hard to give universally accepted definitions of elasto-acoustic metamaterials and PnCs and the differences between them. We subscribe to the definition that metamaterials are structures composed of small meta-atoms with the ability to manipulate waves at frequencies where their wavelengths are much larger than the dimensions of the structural units; furthermore, the metamaterials are not necessarily periodic structures [1,2,3,4,5,6]. In contrast, PnCs can be used to tune the wave propagation at frequencies where the wavelength is of comparable length to their periodicities [7,8,9,10,11,12]. PnCs are widely accepted as periodic structures. Given the definition above, the presented nonperiodic rainbow structures are defined as metamaterials in this paper.

Elasto-acoustic metamaterials are developed from their counterpart of optical and electromagnetic metamaterials, which tailor optical and electromagnetic waves with unusual properties, such as negative magnetic permeability and a negative refractive index. Analogously, the elasto-acoustic metamaterials possess negative mass/dynamic stiffness [13,14,15], negative bulk modulus [16,17], and double negativity [18,19]. One of the most distinguishing properties of metamaterials is the existence of a bandgap, which is a range of frequencies between specified limits through which an electrical or mechanical circuit does not allow signals to pass, or the attenuation is above the required stopband attenuation level. The bandgaps are mainly generated by two mechanisms: Bragg scattering and local resonance. The Bragg scattering bandgaps refer to spectral bands, within which destructive interference of transmitted and scattered waves occurs; hence, the Bragg scattering bandgaps require the wave vector to be 2π/λ, where λ is the dimension of the units in PnCs. By contrast, local resonance bandgaps are caused due to the resonance of internal oscillators. The frequencies of local resonance bandgaps could thus be much lower than that of Bragg scattering bandgaps. Metamaterials and PnCs have potential applications in many fields due to the existence of bandgaps, such as waveguiding and localization [20,21,22,23], sensors [24,25,26], wave collimation and refraction [27,28], etc. In particular, the bandgaps with broadband vibration attenuation could be of vital importance for the vibration and noise control in industries [29,30,31,32].

Metamaterials have received much attention in the past two decades. Despite more and more PnCs and metamaterials of various configurations being proposed and investigated, broadening of the bandwidths remains a challenging but critical issue for both academics and industries. This situation led to the emergence of rainbow metamaterials, which have nonperiodic units. Zhu et al. [33] developed acoustic rainbow trapping that consisted of gradient grooves of different depths, which could trap broadband acoustic waves. Celli et al. [34] found out the effect of bandgap widening by a spatial gradient and the disorder of resonators in rainbow metamaterial plates through experiments. Recently, Meng et al. [35,36] proposed rainbow metamaterial beams with spatially varying cantilever mass oscillators, which possessed broadened one-dimensional bandgaps within multiple frequency ranges compared with periodic beams. These one-dimensional rainbow structures open new avenues for the design of PnCs and metamaterials with enhanced properties. Motivated by this idea, we proposed for the first time a two-dimensional (2D) rainbow metamaterial for the purpose of broadening bandgaps with the hope to excite researchers toward developing efficient analytical models for predicting the wave propagation and frequency response for rainbow structures of higher dimensions. The proposed metamaterials are composed of cubic blocks distributed in 2D space and are connected by symmetrically curved beams. A rainbow metamaterial with a gradient profile is generated by tessellating voids with different dimensions into the blocks. The 2D metamaterials were manufactured using additive manufacturing (AM) technology. The rainbow metamaterial was found to have broader bandgaps at low frequencies compared with the periodic metamaterial using numerical simulations and experiments.

This paper is structured as follows: Section 2 introduces the design of the metamaterials using AM technologies, Section 3 describes the numerical investigation on the frequency response functions (FRFs) and mode shapes of the metamaterials, and Section 4 presents the tested results and the discussion. Conclusions are drawn in Section 5.

## 2. Design of 2D Rainbow Metamaterials

**Figure 1:**
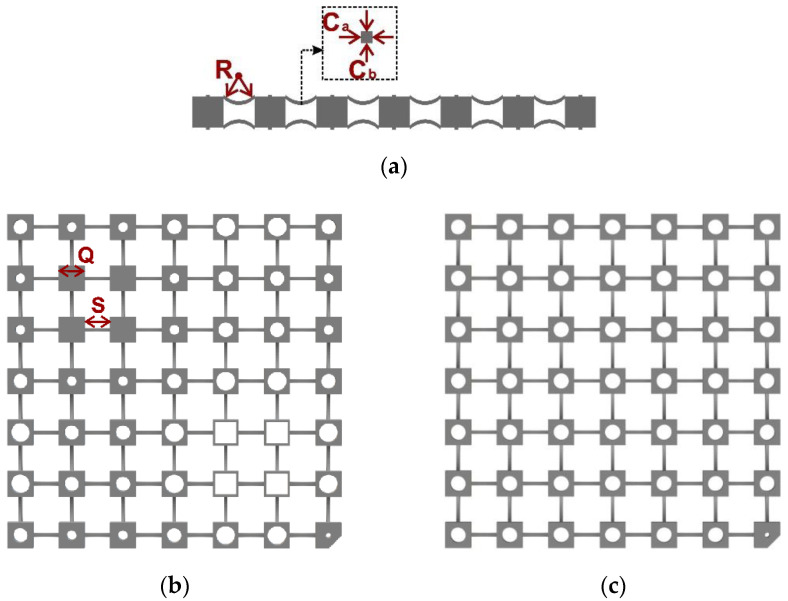
Schematic diagrams of the 2D rainbow and periodic metamaterials: (**a**) side view, (**b**) top view of the rainbow metamaterial, and (**c**) top view of the periodic metamaterial. The labels show the geometric parameters: block side length (*Q*), distance between blocks (*S*), curvature radius of the curved beam (*R*), and cross-section dimensions of the curved beams (*C_a_*, *C_b_*).

For the purpose of delivering the rainbow design, metamaterials with distributed mass blocks in 2D space were designed as shown in Figure 1a–c. Internal voids of different dimensions were incorporated into the blocks in a rainbow metamaterial, which hence had varied masses, as can be seen in Figure 1b. It is noted that the minimum block mass was set to 30% of that of blocks without voids, which can maintain the intact external shapes of the blocks, as well as comply with the assumption of the blocks as rigid bodies. Two inverted symmetric curved beams were inserted between blocks as connection components. The curved beams could increase the stability and decrease the transmission stiffness compared with straight ones. The distance between the blocks was *S* = 20 mm, the side length of cubic blocks was *Q* = 20 mm, and the sectional dimensions of the curved beams were *C_a_* = *C_b_* = 2.4 mm. The masses of the blocks in the rainbow metamaterial were assumed to be distributed sinusoidally in the *x*- and *y*-directions according to:

(1) $$m_{ij}=\left(0.74+0.19\sin\left(\frac{\left(i-1\right)\pi }{3}\right)+0.19\sin\left(\frac{\left(j-1\right)\pi }{3}\right)\right)M$$

where *M* is the mass of blocks without voids, and *i*, *j* = 1, 2, …, 7 represent the block numbers in the *x*- and *y*-directions. Sinusoidal distributions can not only introduce a gradually changing profile but more complex distributions could easily be created on the basis of simple sinusoidal distributions. It is noted that although the bandgaps can be enlarged with the increase of the block number, the requirements for manufacturing and numerical simulation would also be greatly improved. Seven unit cells in each direction were therefore adopted in the present study, which could display dramatic bandgaps, as well as comply with the limitations of manufacturing and numerical simulation. An equivalent metamaterial with the same mass as the rainbow one was also proposed by tessellating identical voids into the blocks, as shown in Figure 1c. The volume of the periodic voids was equal to the average void volume in the rainbow metamaterial, e.g., *m_ij_* = 0.74 *M*.

## 3. Numerical Modelling of the Metamaterials

The receptance functions (i.e., displacement FRFs) and modal shapes of the presented periodic and rainbow metamaterial structures were calculated using a finite element (FE) modeling method. Owing to the non-periodicity of the rainbow metamaterial, the complete metamaterial instead of a unit cell was modeled in the FE models. The FE models were set up using the solid mechanics module in Comsol Multiphysics. Excitation forces in *x*- and *y*-directions were exerted on one corner of the metamaterials, and the displacements at the other corner were computed, such that the receptance functions (i.e., displacement FRFs) could finally be calculated based on the excitation forces and displacements using:

(2) $$R_{x}=20\log_{10}\left|D_{x}/F_{x}\right|,R_{y}=20\log_{10}\left|D_{y}/F_{y}\right|,$$

where *F_x_* and *F_y_*, and *D_x_* and *D_y_* are the tested forces and displacements in the *x*- and *y*-directions. The material of the metamaterials was assumed to have a density of 1010 kg/m^3^, a loss factor of 0.03, and a Young’s modulus of 1.64 GPa, which are the parameters of structures manufactured using additive manufacturing with PA12 powder [37].

**Figure 2:**
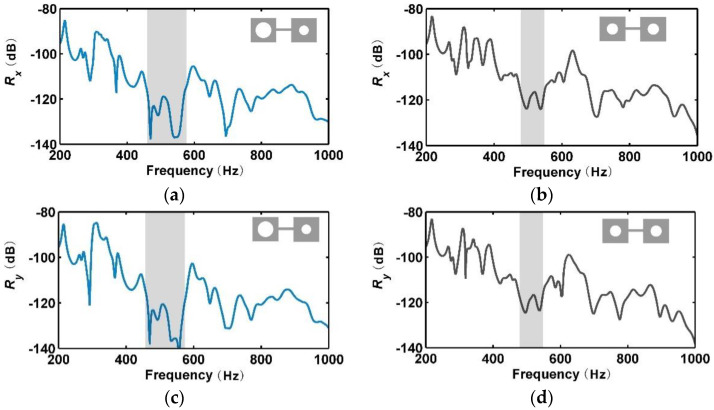
Receptance functions of the rainbow metamaterial in the *x*- (**a**) and *y*- (**c**) directions, and of the periodic metamaterial in the *x*- (**b**) and *y*- (**d**) directions from numerical simulations. The bandgaps are marked in grey.

**Figure 3:**
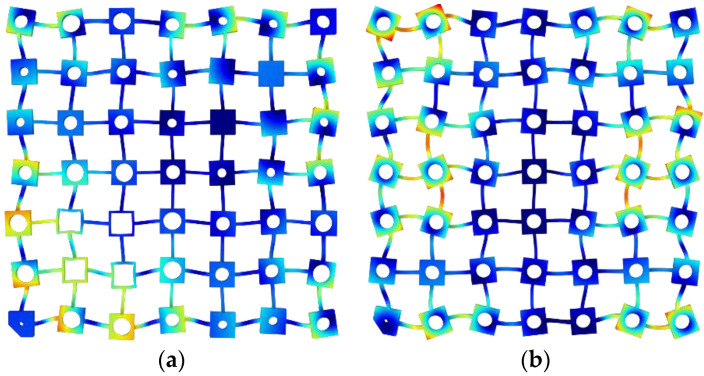

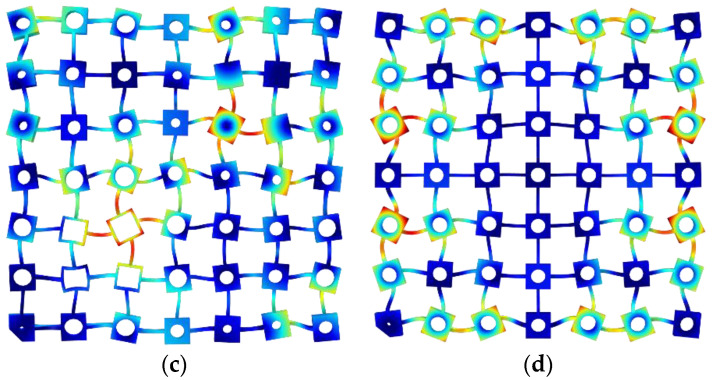
Mode shapes of the metamaterials at the start and end frequencies of the bandgaps: the rainbow metamaterial at (**a**) 444 Hz and (**c**) 578 Hz, and the periodic metamaterial at (**b**) 466 Hz and (**d**) 549 Hz.

The receptance functions of the metamaterials in the *x*- and *y*-directions are shown in Figure 2a–d. It can be seen that the periodic metamaterial had a dramatic vibration attenuation band in the frequency range of 466–549 Hz, as marked in grey in Figure 2b,d. The ratio of the bandwidth to the center frequency of the bandgap was around 16.3%. In contrast, the rainbow metamaterial possessed dramatic attenuation over the frequency range of 444–578 Hz, which had a bandwidth-to-center-frequency ratio of 26.2%. The mode shapes of the two metamaterials at the starting and ending frequencies of the bandgap are shown in Figure 3a–d. The bandgap of the rainbow metamaterial was hence more than 60% wider than that of the periodic metamaterial of the same mass. Given that wavelengths at the bandgap frequencies are much larger than the lattice dimension of metamaterials, the bandgaps should be attributed to the local resonance. This has also been proved by Achaoui et al. [38] that different from local resonance bandgaps which could be tuned by the disorder of units in metamaterials, the Bragg bandgaps would disappear with it. As the resonance and bandgap frequencies change with the mass of blocks, the variation of blocks in a rainbow metamaterial would hence result in enlarged bandgaps.

## 4. Experimental Measurements

**Figure 4:**
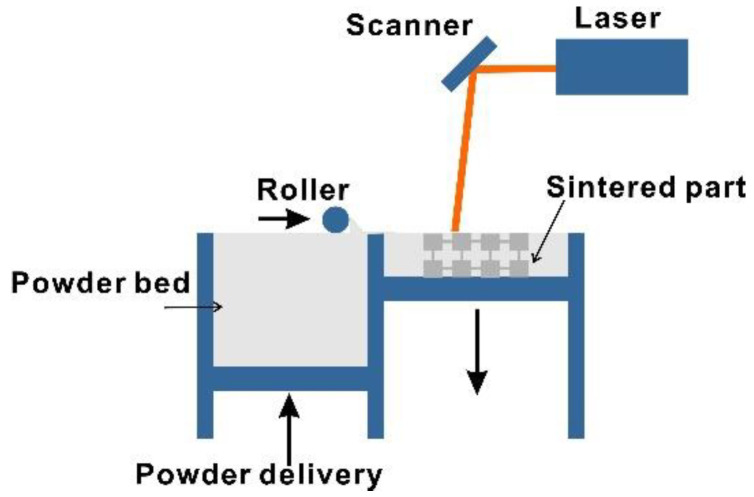
Schematic illustration of the selective laser sintering system.

In order to further explore the influences of the rainbow design and validate the above numerical results, experimental measurements were conducted and the results are presented in this section. Two rainbow and periodic metamaterials samples were manufactured using the AM method. Advanced manufacturing technologies, especially AM, allow for the fabrication of complex parts with high precision at a lower cost; hence, they could undoubtedly facilitate the development of novel metamaterials. AM is one of the most popular methods for the fabrication of metamaterials and metamaterials. There are a lot of AM technologies that vary in their materials and layer depositing processes, including material jetting, binder jetting, stereolithography, selective laser sintering (SLS), etc. [39,40]. The SLS technology creates parts by sintering the powder layer-by-layer with a laser, as shown schematically in Figure 4. Compared with other technologies, SLS is best for the rapid fabrication of strong parts with complex geometries without the requirement of extra support; hence, it was selected to manufacture the 2D metamaterials in the study. The structures were manufactured using PA12 powder filled in a powder bed of 375 mm× 375 mm× 430 mm. A CO_2_ laser was employed to provide the sintering power with a nominal spot size of 0.46 mm and a layer thickness of 0.12 mm. The precision of the manufactured structures can be affected by the laser spot size, the layer thickness, the laser scanning strategy, etc. Features with dimensions below 1 mm could have considerable discrepancies in the mechanical parameters owing to unsolidified powder [41].

**Figure 5:**
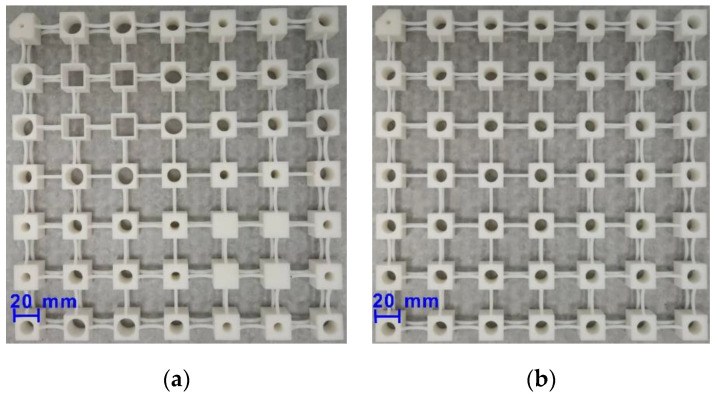
Photographs of the manufactured (**a**) rainbow metamaterial and (**b**) periodic metamaterial using selective laser sintering (SLS).

**Figure 6:**
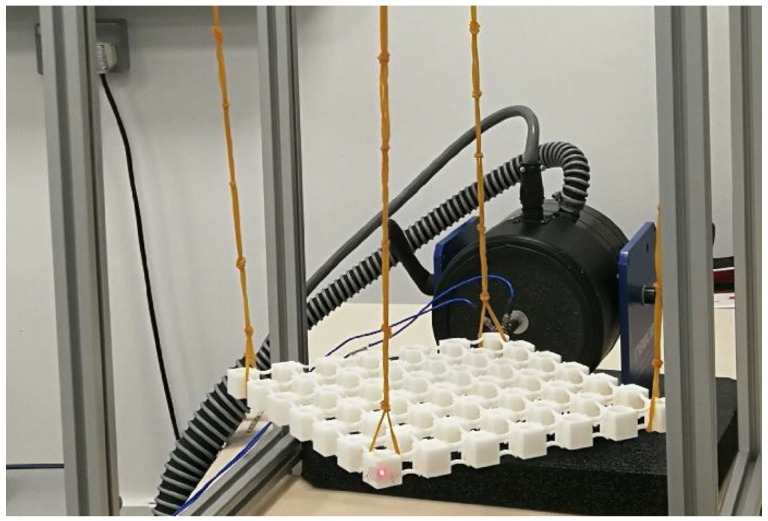
Photograph of the experimental setup for the frequency response functions (FRFs) test of the 2D metamaterial.

**Figure 7:**
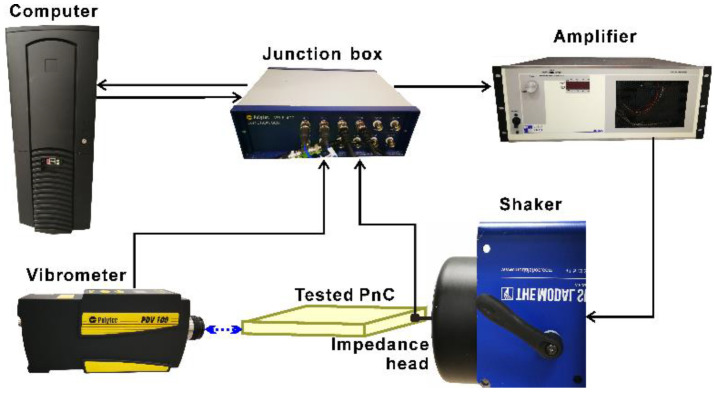
Illustration of the full experimental system. PnC: phononic crystal.

Photographs of the two printed metamaterials are shown in Figure 5a,b. The FRFs of the metamaterials were measured by the experimental system, as shown in Figure 6. The metamaterials were suspended from a metal frame with rubber bands during the experiment, which could compensate for gravity in the direction perpendicular to the metamaterials. Suspension of tested structures has been proved to be an effective approach to simulating the free boundary conditions for the testing of PnCs and metamaterials [1,42,43]. An illustration of the full experimental system can be seen in Figure 7. The tested metamaterials were glued to a mechanical shaker (Modal Shop 2060E, Modal Shop, Cincinnati, OH, USA) at one vertex and excited by chirp waves in the frequency range of 200–1000 Hz. The excitation signal was first generated using a computer and a junction box (Polytec VIB-E-400, Polytec, Coventry, UK) and later amplified by an amplifier (Modal Shop 2050E09 amplifier, Modal Shop, Cincinnati, OH, USA). An impedance head (PCB 288D01, PCB, Depew, NY, USA) was employed to measure the actual forces exerted on the metamaterial, and a Doppler vibrometer (Polytec PSV-400, Polytec, Coventry, UK) was employed to measure the displacements at the diagonal vertex in two directions. Both measurements were conducted with a frequency resolution of 1.25 Hz and averaged over 100 sweeps. The tested forces and displacements were collected by the junction box and sent back to the computer afterward.

**Figure 8:**
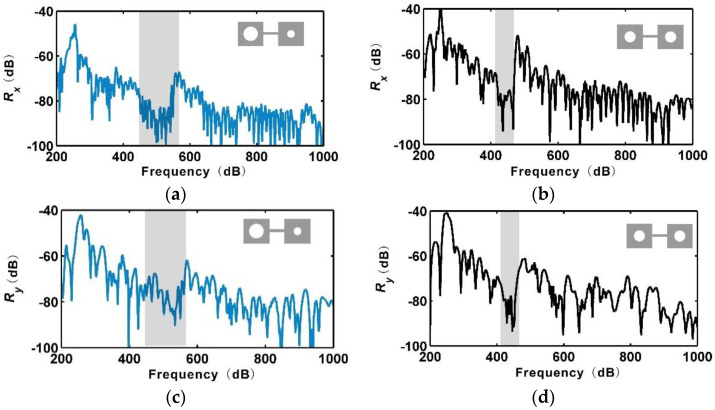
Experimental receptance functions of the rainbow metamaterial in the *x*- (**a**) and *y*- (**c**) directions, and of the periodic metamaterial in the *x*- (**b**) and y- (**d**) directions. The bandgaps are marked in grey.

The measured FRFs of the two metamaterials are shown in Figure 8a,d. It can be seen that the experimental results show the same trend as the numerical results. The rainbow metamaterial possessed a bandgap of approximately 460–550 Hz, which was wider than that of the periodic metamaterial of approximately 420–470 Hz. The receptance functions within the bandgaps were reduced by around 15 dB. It is noted that reasonable discrepancies lay in the bandgap frequencies and receptance values of the numerical and experimental results, which were caused by the non-ideal experimental conditions and uncertainties introduced by the AM process. It was found that AM could lead to variabilities in the elastic modulus and density of up to more than 10%, where these variabilities would remarkably alter the bandgap frequencies and FRFs of the metamaterials [37,44]. The errors of the loss factor and geometric parameters of the manufactured structures could have also contributed to the discrepancies. Regardless of the reasonable discrepancies, both the experiments and numerical simulations showed the effectiveness of broadening the bandgaps using a nonperiodic design.

## 5. Conclusions

In this study, the vibration attenuation performance of 2D rainbow metamaterials were investigated numerically and experimentally. The metamaterials were composed of spatially distributed blocks connected by curved beams. Internal voids were implemented in the blocks to adjust the local masses and generate nonperiodic profiles. The rainbow metamaterial contained blocks with masses that varied sinusoidally in two directions. The receptance functions of the developed metamaterials were calculated using FE models and measured using an experimental system. It was found via the experiment that the rainbow metamaterial had low-frequency bandgaps that were much broader than periodic metamaterials of equal mass. The method of introducing nonperiodicity shows great potential for the design of metamaterials with enhanced attenuation performance.
